# Short-Term Mild Hypoxia Modulates Na,K-ATPase to Maintain Membrane Electrogenesis in Rat Skeletal Muscle

**DOI:** 10.3390/ijms231911869

**Published:** 2022-10-06

**Authors:** Violetta V. Kravtsova, Arina A. Fedorova, Maria V. Tishkova, Alexandra A. Livanova, Viacheslav O. Matytsin, Viacheslav P. Ganapolsky, Oleg V. Vetrovoy, Igor I. Krivoi

**Affiliations:** 1 Department of General Physiology, Saint Petersburg State University, 199034 Saint Petersburg, Russia; 2 Department of Pharmacology and Pharmacy, Mechnikov North-Western State Medical University, 191015 Saint Petersburg, Russia; 3 Pavlov Institute of Physiology, Russian Academy of Sciences, 199034 Saint Petersburg, Russia; 4 Department of Biochemistry, Saint Petersburg State University, 199034 Saint Petersburg, Russia

**Keywords:** skeletal muscle, hypobaric hypoxia, Na,K-ATPase isozymes, resting membrane potential, endogenous ouabain

## Abstract

The Na,K-ATPase plays an important role in adaptation to hypoxia. Prolonged hypoxia results in loss of skeletal muscle mass, structure, and performance. However, hypoxic preconditioning is known to protect against a variety of functional impairments. In this study, we tested the possibility of mild hypoxia to modulate the Na,K-ATPase and to improve skeletal muscle electrogenesis. The rats were subjected to simulated high-altitude (3000 m above sea level) hypobaric hypoxia (HH) for 3 h using a hypobaric chamber. Isolated diaphragm and soleus muscles were tested. In the diaphragm muscle, HH increased the α2 Na,K-ATPase isozyme electrogenic activity and stably hyperpolarized the extrajunctional membrane for 24 h. These changes were accompanied by a steady increase in the production of thiobarbituric acid reactive substances as well as a decrease in the serum level of endogenous ouabain, a specific ligand of the Na,K-ATPase. HH also increased the α2 Na,K-ATPase membrane abundance without changing its total protein content; the plasma membrane lipid-ordered phase did not change. In the soleus muscle, HH protected against disuse (hindlimb suspension) induced sarcolemmal depolarization. Considering that the Na,K-ATPase is critical for maintaining skeletal muscle electrogenesis and performance, these findings may have implications for countermeasures in disuse-induced pathology and hypoxic therapy.

## 1. Introduction

Skeletal muscle, which is one of the largest tissues in the body, is important for general health and wellness and adapts to pathophysiological conditions with amazing plasticity. Skeletal muscle activity is essential to maintain both muscle mass and muscle function. Conversely, physical inactivity results in muscle wasting and atrophy [1,2].

The supply of oxygen to cells and tissues is of paramount importance for their function and survival. Hypoxia is a universal process that manifests itself in a number of pathophysiological conditions [3]. The effect of hypoxia on muscle mass and function is less investigated compared to inactivity. Several studies have reported that prolonged exposure to hypoxia (in response to various pathological and physiological conditions including high altitude) results in loss of muscle mass, structure, and performance [4,5,6,7]. Moreover, hypoxia aggravates inactivity-related muscle wasting [8]. Recent studies show that pulmonary dysfunction in COVID-19 infection is also characterized by impaired oxygen homeostasis and hypoxia [9], as well as dysfunction of the diaphragm muscle [10,11]. Notably, respiratory muscle dysfunction adversely affects survival, and its remodeling plays a vital physiological role. However, despite the fact that the diaphragm is the main respiratory muscle, relatively little research has been focused on its hypoxia-related plasticity [12,13,14,15].

Among different mechanisms involved in maintaining sarcolemmal electrogenesis and skeletal muscle performance, the activity of Na,K-ATPase is critically important [16,17,18] and the Na,K-ATPase plays an important role in adaptation to hypoxia [4,19].

The Na,K-ATPase is a heteromeric complex consisting of a catalytic and transport α subunit and glycoprotein β subunit. Four isoforms of the α subunit and three isoforms of the β subunit are expressed in a cell- and tissue-specific manner [20,21,22,23,24]. Skeletal muscles co-express the α1 and α2 isozymes of Na,K-ATPase [25,26]. While the α1 Na,K-ATPase isozyme is relatively uniformly distributed in the sarcolemma, there are two distinct pools (extrajunctional and junctional) of the α2 isozyme. The majority of extrajunctional α2 Na,K-ATPase isozyme is expressed in the interior transverse tubule membranes [27]. The smaller α2 Na,K-ATPase pool is localized to the junctional (endplate) membrane, where it interacts with the nicotinic acetylcholine receptors (nAChRs) to regulate the resting membrane potential (RMP) [28]. The extracellular loops of the Na,K-ATPase α subunit form a specific binding site for cardiotonic steroids of plant and animal origin which have endogenous analogs in mammalian tissues and circulation. Among these ligands, ouabain is regarded as a hormone with the Na,K-ATPase as a receptor [24,29,30,31,32].

Steady membrane depolarization due to loss of the α2 Na,K-ATPase function is characteristic of motor dysfunction including both chronic [33] and acute [34,35] skeletal muscle disuse. This depolarization is among the earliest remodeling events induced by postural soleus muscle disuse [35] and is suggested to be responsible for the accumulation of intracellular calcium that might be a key trigger to downstream signaling events leading to muscle atrophy [36,37]. Functional unloading leads to skeletal muscle atrophy, which begins to appear after 2–3 days of disuse [1,2]. The mechanisms of disuse-induced atrophy are numerous and remain largely obscure [1,2,37,38]. From the point of view of the search for key signaling events that trigger the atrophic program, the mechanisms of the early stages of disuse preceding the development of overt atrophy are of great interest [37].

Therefore, the search for countermeasures to improve membrane electrogenesis could have promising therapeutic implications. It is desirable that these preventive approaches be non-invasive and do not involve pharmacological intervention. Considering that hypoxic preconditioning has preventive effects against a number of functional disorders such as ischemic injury [39,40,41] and diaphragm muscle dysfunction [13,15] we opted for mild hypoxia.

In this study, we hypothesized that short-term mild hypoxia is able to modulate the Na,K-ATPase to maintain skeletal muscle electrogenesis, a subject not previously addressed. The rats were subjected to hypobaric hypoxia (HH) at a pressure equivalent to an altitude of 3000 m above sea level for 3 h using a hypobaric chamber. The isolated diaphragm and soleus muscles were investigated using biochemical assay, conventional microelectrode technique, as well as Western blotting and confocal microscopy with cytochemistry.

## 2. Results

### 2.1. Hypobaric Hypoxia Decreases the Serum Level of Endogenous Ouabain and Increases the Production of TBARS

**Figure 1:**
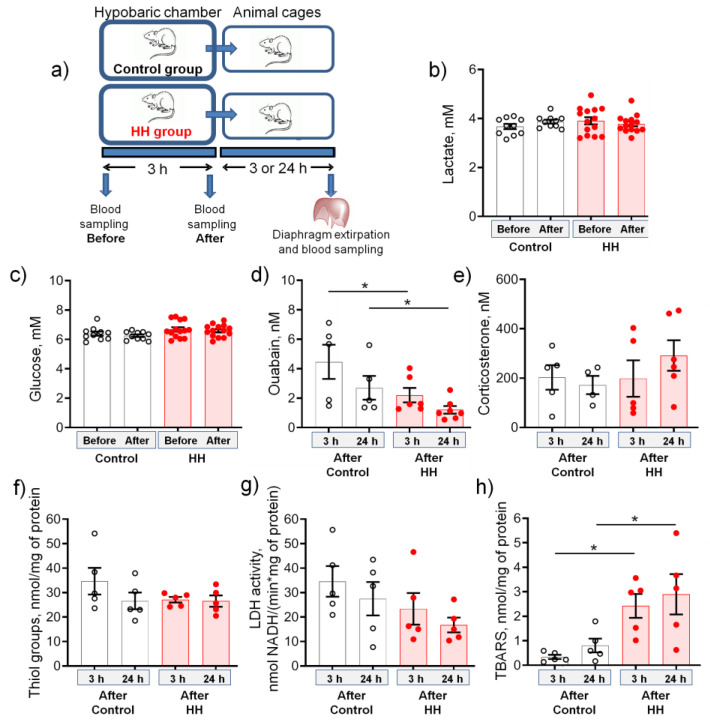
Biochemical responses to hypobaric hypoxia (HH) for 3 h at a pressure equivalent to an altitude of 3000 m. (**a**) The study outline. (**b**,**c**) Blood lactate and glucose concentrations measured immediately before and after control or HH treatment. (**d**,**e**) Concentrations of ouabain and corticosterone measured in the blood serum 3 or 24 h after control or HH treatment. (**f**) The concentration of thiol groups, (**g**) lactate dehydrogenase (LDH) activity, and (**h**) thiobarbituric acid reactive substances (TBARS) concentration measured in homogenate of the diaphragm muscle 3 or 24 h after control or HH treatment. The number of rats corresponds to the number of symbols. In these experiments, two groups of 10–14 rats of each category (control and HH, respectively) were in the pressure chamber for 3 h, and their characteristics are provided in **b** and **c**. Then, the rats from each of these categories were divided into two groups (5–7 rats), which were kept in cages for 3 or 24 h for further analysis (**d**–**h**). Two-way ANOVA followed by Bonferroni multiple comparisons test. * *p* < 0.05—compared to the corresponding control.

The study design is presented in Figure 1a. Exposure to HH for 3 h did not affect both lactate and glucose levels, measured in the rat blood immediately before and after HH (Figure 1b,c). Exposure to HH significantly (*p* < 0.05) decreased serum ouabain level within 24 h after HH (Figure 1d). Thus, in contrast to prolonged (3 days) experimental hypoxia, which increased the release of endogenous ouabain [42], short-term (3 h) exposure to HH decreases serum ouabain level. Serum corticosterone, which is often used as a biomarker of stress and cross-talked with hypoxic signaling [43], has not changed significantly (Figure 1e).

Hypobaric hypoxia did not affect the number of reduced thiol groups in cytosolic proteins used as a marker of cellular redox status, as well as lactate dehydrogenase (LDH) activity measured in the diaphragm muscle homogenate (Figure 1f,g). At the same time, thiobarbituric acid reactive substances (TBARS) production was steadily increased within 24 h after HH (Figure 1h) suggesting an increased level of lipid peroxidation.

In sum, no pronounced signs of stress were found, which emphasizes the mild nature of the hypoxic state in this study.

### 2.2. Hypobaric Hypoxia Hyperpolarizes Diaphragm Sarcolemma due to a Steady Increase in the α2 Na,K-ATPase Electrogenic Activity

**Figure 2:**
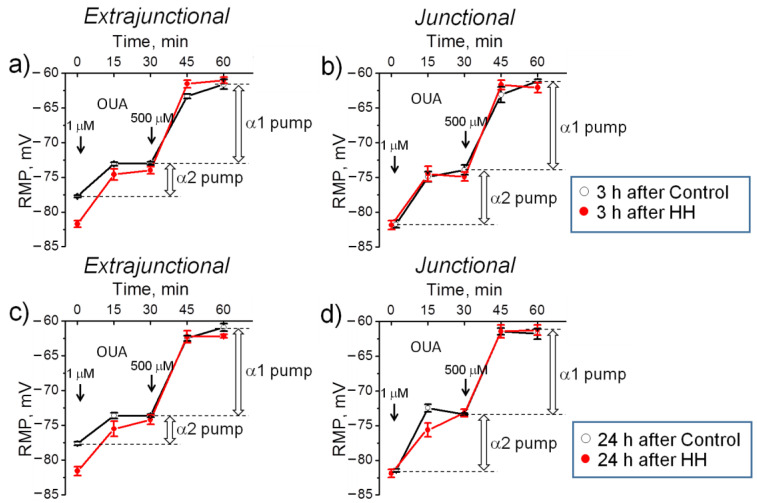
Changes in the resting membrane potential (RMP) of diaphragm muscles isolated from rats subjected to hypobaric hypoxia (HH) at a pressure equivalent to an altitude of 3000 m for 3 h. The measurements were carried out on muscles isolated 3 (**a**,**b**) or 24 h (**c**,**d**) after control or HH treatment in the extrajunctional (**a**,**c**) and junctional (**b**,**d**) sarcolemmal regions. The electrogenic contribution of the α2 and α1 Na,K-ATPase was estimated by administration of 1 or 500 µM ouabain (OUA, indicated by the corresponding arrows). Broad vertical arrows indicate electrogenic contributions generated by the α1 and α2 Na,K-ATPase isozymes in control muscles. Each data point corresponds to the averaged RMP measured in 5 muscles.

The measurements of RMP were performed on diaphragm muscles isolated from rats 3 or 24 h after exposure to HH. The transport activity of the Na,K-ATPase α1 and α2 isozymes was determined by measuring the ouabain-sensitive changes in RMP (see Methods). For each muscle, the initial RMP value was recorded (time ‘zero’ in Figure 2). Then ouabain was sequentially added to the solution at concentrations of 1 and 500 μM. The electrogenic contribution of α2 isozyme was computed as the difference of RMP before and 30 min after the incubation with 1 µM ouabain. Then, the electrogenic contribution of the α1 isozyme was estimated as the difference in RMP with 1 µM ouabain and after 30 min incubation with 500 µM ouabain (Figure 2a–d).

**Figure 3:**
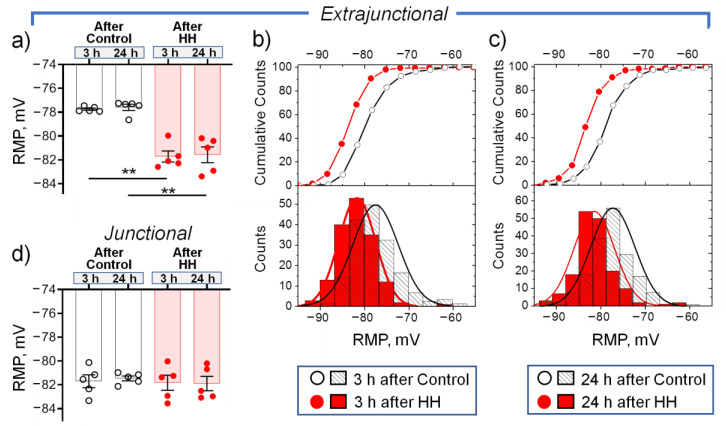
Changes in the resting membrane potential (RMP) of diaphragm muscles isolated from rats subjected to hypobaric hypoxia (HH) at a pressure equivalent to an altitude of 3000 m for 3 h. The measurements were carried out on muscles isolated 3 or 24 h after control or HH treatment in the extrajunctional (**a**–**c**) and junctional (**d**) sarcolemmal regions. (**a,d**) The number of muscles corresponds to the number of symbols. (**b,c**) Cumulative probability curves and corresponding histograms of RMP distributions 3 (**b**) or 24 h (**c**) after control or HH; the same muscles as in (**a**). The total number of RMP measurements (control/HH): (**b**) 156/159; (**c**) 161/167. Two-way ANOVA followed by Bonferroni multiple comparisons test. ** *p* < 0.01—compared to the corresponding control.

In these experiments, 3 h after HH, extrajunctional membrane hyperpolarized from −77.8 ± 0.2 mV in control muscles to −81.7 ± 0.5 mV (*p* < 0.01) in hypoxia-treated muscles. Thus, the extrajunctional region was significantly hyperpolarized with –3.9 ± 0.6 mV (Figure 3a). Accordingly, corresponding cumulative probability curves as well as histograms of RMP distribution in hypoxia-treated muscles were shifted to more negative values (Figure 3b). This hyperpolarizing effect persisted for 24 h after HH (*p* < 0.01) (Figure 3a,c). At the same time, no significant changes in the RMP were observed in the junctional membrane region (Figure 3d). This observation is in accordance with a previous report on differential regulation of the extrajunctional and junctional membrane regions in various interventions [35,44].

**Figure 4:**
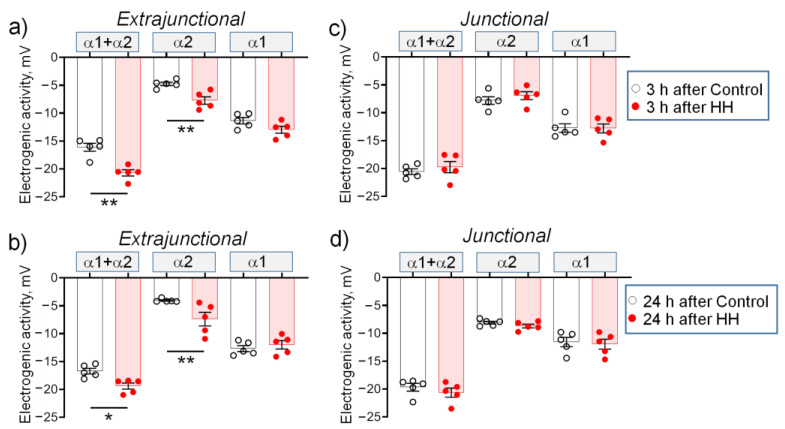
(**a**–**d**) Electrogenic activity of the α2 and α1 Na,K-ATPase isozymes in diaphragm muscles isolated from rats subjected to hypobaric hypoxia (HH) at a pressure equivalent to an altitude of 3000 m for 3 h. The measurements were carried out on muscles isolated 3 or 24 h after control or HH treatment. The electrogenic contribution of the α2 and α1 Na,K-ATPase was estimated by administration of 1 or 500 µM ouabain (as shown in Figure 2a–d). The number of muscles corresponds to the number of symbols. The measurements were carried out in extrajunctional (**a**,**b**) and junctional (**c**,**d**) sarcolemmal regions. Two-way ANOVA followed by Bonferroni multiple comparisons test. * *p* < 0.05, ** *p* < 0.01— compared to the corresponding control.

In the extrajunctional membrane region of control muscles, 3 h after HH, total (α2 + α1 isozyme) electrogenic activity by the Na,K-ATPase contributes to the RMP with –16.1 ± 0.7 mV. Of this, the α2 isozyme generates –4.7 ± 0.3 mV and the α1 isozyme generates –11.4 ± 0.6 mV (Figure 4a). Exposure to HH significantly (*p* < 0.01) increased total Na,K-ATPase contribution to −20.6 ± 0.5 mV. The α2 isozyme contribution increased to −7.8 ± 0.7 mV (*p* < 0.01) while the α1 isozyme contribution remained unchanged (Figure 4a). These measurements suggest that HH-induced membrane hyperpolarization is mediated by increased electrogenic activity of the Na,K-ATPase and that the α2 isozyme is preferentially modulated. Similar changes were observed 24 h after HH (Figure 4b). 

In the junctional membrane region, no HH-dependent changes were observed (Figure 4c,d). Notably, the junctional membrane region of control muscles was initially hyperpolarized vs. the extrajunctional membrane of the same muscles by −3.9 ± 0.6 mV (*p* < 0.01 (−81.7 ± 0.5 mV vs. −77.8 ± 0.2 mV, respectively) (Figure 3a,d). Such local hyperpolarization is attributed to enhanced electrogenic activity of the Na,K-ATPase α2 isozyme at the neuromuscular junction of resting muscles [28]. This is confirmed in this study: in the junctional membrane of control muscles, the α2 isozyme generates –7.8 ± 0.7 mV vs. –4.7 ± 0.3 mV (*p* < 0.01) in the extrajunctional membrane (Figure 4a,c). This initial increased activity of the α2 isozyme pool may be the reason for the inability of HH to further activate it and to hyperpolarize the RMP.

**Figure 5:**
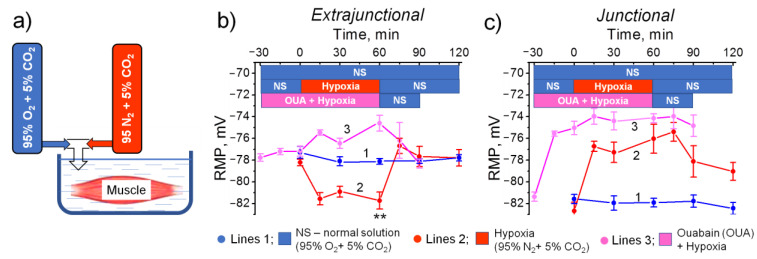
(**a**) The study outline. Acute effects of hypoxia on the RMP at the extrajunctional (**b**) and junctional (**c**) membrane regions of the isolated rat diaphragm muscle. The RMP dynamics of muscles perfused with different solutions are shown. The muscles were exposed to 60 min hypoxia (95 N_2_ + 5% CO_2_) followed by 60 min reoxygenation in the normal physiological solution (NS, 95% O_2_ + 5% CO_2_). In the control muscles, RMPs were recorded in the normal solution within 120 min. Ouabain (50 nM) incubation was started 30 min prior to adding the hypoxic solution. Each data point corresponds to the averaged RMP measured in 5–7 muscles. One-way ANOVA followed by Bonferroni multiple comparisons test. ** *p* < 0.01—compared to the corresponding control point.

In separate experiments, diaphragm muscles isolated from intact (not subjected to HH) rats were exposed to normobaric hypoxia (Figure 5a). Initial RMPs were recorded in the normal physiological solution bubbled with 95% O_2_ + 5% CO_2_. The muscles were then exposed to 60 min hypoxia (95 N_2_ + 5% CO_2_) followed by 60 min reoxygenation (95% O_2_ + 5% CO_2_), as described previously [15]_._ In the control muscles, RMPs were recorded in the normal solution within 120 min. Similarly to HH, hypoxic solution hyperpolarized the extrajunctional membrane with −3.6 ± 0.8 mV (*p* < 0.01, 60 min hypoxia). Hyperpolarization developed after 15 min and remained stable for the 60 min hypoxia. This hyperpolarization was reversible and reoxygenation with a normal solution returned the RMP to its initial value (Figure 5b).

Preincubation with 50 nM ouabain for 30 min before exposure to normobaric hypoxia completely prevented membrane hyperpolarization in a hypoxic solution (Figure 5b). This suggests that hypoxia-induced hyperpolarization results from stimulated electrogenic transport by the ouabain-sensitive Na,K-ATPase α2 isozyme.

In the junctional membrane region, only membrane depolarization was observed (Figure 5c).

### 2.3. Hypobaric Hypoxia Increases the α2 Na,K-ATPase Membrane Abundance without Changing its Total Protein Content

**Figure 6:**
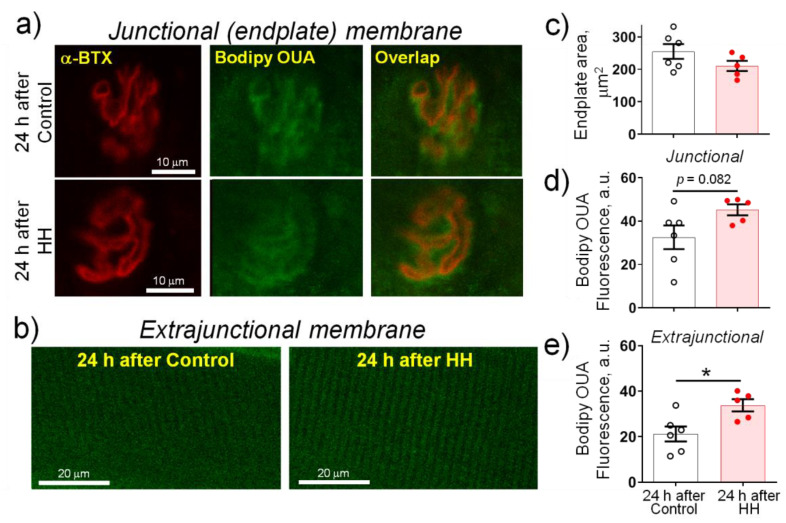
Exposure to hypobaric hypoxia (HH) at a pressure equivalent to an altitude of 3000 m for 3 h increases the α2 Na,K-ATPase membrane abundance in the rat diaphragm muscle. The measurements were carried out on muscles isolated 24 h after control or HH treatment. (**a**) Representative images of endplates dual-labeled with α-BTX (nAChRs, red channel) and Bodipy-conjugated ouabain (α2 Na,K-ATPase, green channel). Overlap—orange. Scale bars—10 μm. (**b**) Representative images of extrajunctional membrane regions. Scale bars—20 μm. (**c**) Averaged area of endplates. (**d**,**e**) Averaged fluorescence intensity from the α2 Na,K-ATPase staining (arbitrary units) in the junctional (**d**) and extrajunctional (**e**) membrane regions. The number of muscles corresponds to the number of symbols. One-way ANOVA followed by Bonferroni multiple comparisons test. * *p* < 0.05—compared to the corresponding control.

To test the membrane localization of α2 Na,K-ATPase, we investigated images of diaphragm muscles, which were dually labeled with Bodipy-conjugated ouabain and the nAChRs (rhodamine-conjugated α-BTX) (see Methods). Both junctional (endplate) (Figure 6a) and extrajunctional (Figure 6b) membrane regions were examined 24 h after control or HH treatment. Fluorescent signals from the nAChRs and the α2 Na,K-ATPase overlaps at the endplate regions, as previously reported [28]. The endplates area of the control muscles was 255 ± 23 μm^2^ and did not change significantly after HH (Figure 6c). The fluorescence intensity from the α2 Na,K-ATPase in the endplate membrane regions showed a pronounced tendency to increase (*p* = 0.082) (Figure 6d) and was significantly increased (*p* < 0.05) in the extrajunctional membrane regions (Figure 6e), suggesting an increased density of distribution of this protein in the sarcolemma.

**Figure 7:**
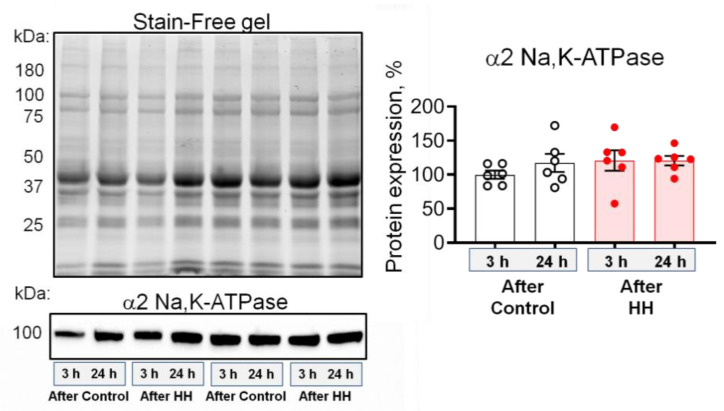
Exposure to hypobaric hypoxia (HH) at a pressure equivalent to an altitude of 3000 m for 3 h does not affect the total α2 Na,K-ATPase content in the rat diaphragm muscle. The measurements were carried out on muscles isolated 3 or 24 h after control or HH treatment. Left —representative total protein load detected with Stain-Free gel and Western blots for the α2 Na,K-ATPase, used for semi-quantification of its protein expression. Right—the averaged results of semi-quantification of the α2 Na,K-ATPase expression. The number of muscles corresponds to the number of symbols. Two-way ANOVA followed by Bonferroni multiple comparisons test.

Next, we tested whether HH modulates the α2 Na,K-ATPase total protein content measured in homogenates of whole diaphragm muscles and no significant changes were observed (Figure 7). Thus, HH increases the electrogenic activity of α2 Na,K-ATPase, as well as its membrane abundance without changing its total protein content.

### 2.4. Hypobaric Hypoxia Does Not Affect the Plasma Membrane Lipid-Ordered Phase

**Figure 8:**
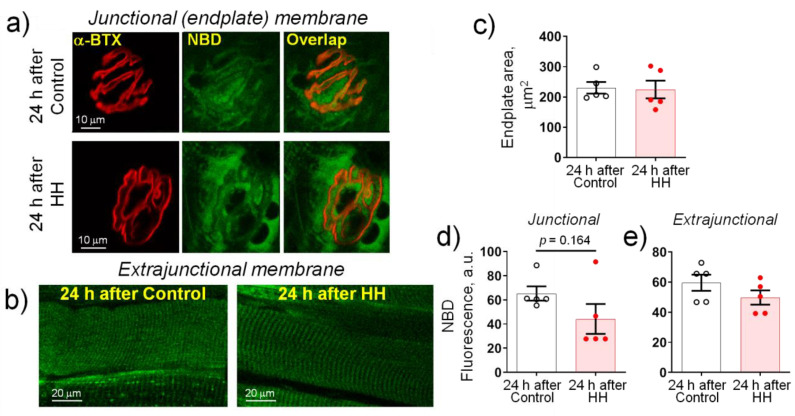
Exposure to hypobaric hypoxia (HH) at a pressure equivalent to an altitude of 3000 m for 3 h does not affect the membrane distribution of fluorescent 22-NBD-cholesterol in the rat diaphragm muscle. The measurements were carried out on muscles isolated 24 h after control or HH treatment. (**a**) Representative images of endplates dual-labeled with α-BTX (nAChRs, red channel) and 22-NBD-cholesterol (NBD, green channel). Overlap—orange. Scale bars—10 μm. (**b**) Representative images of extrajunctional membrane regions. Scale bars—20 μm. (**c**) Averaged area of endplates. (**d**,**e**) Averaged fluorescence intensity from the 22-NBD-cholesterol staining (arbitrary units) in the junctional (**d**) and extrajunctional (**e**) membrane regions. The number of muscles corresponds to the number of symbols. Two-way ANOVA followed by Bonferroni multiple comparisons test.

The activity of Na,K-ATPase is regulated by the lipid environment [45,46,47]. In this study, the integrity of the plasma membrane lipid-ordered phase was tested using fluorescent 22-NBD-cholesterol, whose distribution is opposite to cholesterol [48,49]. Both junctional (endplate) (Figure 8a) and extrajunctional (Figure 8b) membrane regions were examined 24 h after control or HH treatment.

The endplates area did not change significantly after HH (Figure 8c). In the junctional (endplate) membrane regions, the fluorescence intensity from 22-NBD-cholesterol showed only a slight tendency to decrease (*p* = 0.164) (Figure 8d) and did not change significantly in the extrajunctional membrane regions (Figure 8e). This suggests that HH does not affect the plasma membrane lipid-ordered phase.

### 2.5. Hypobaric Hypoxia Protects against Disuse-Induced Disruption of Soleus Muscle Electrogenesis

**Figure 9:**
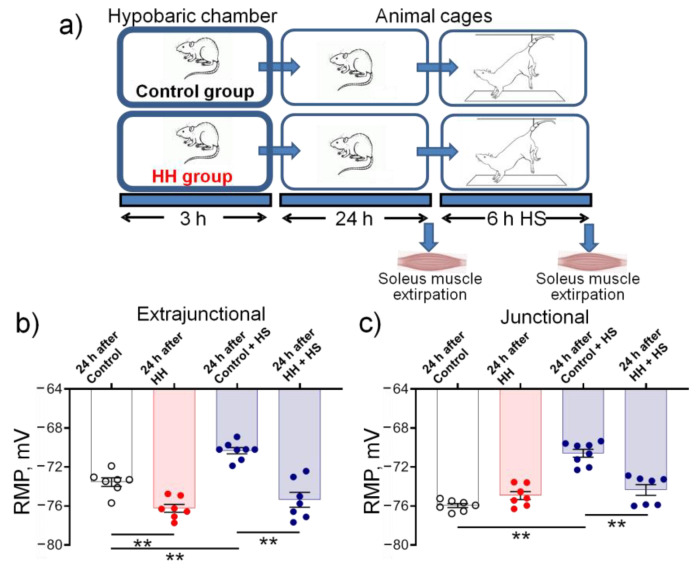
(**a**) The study outline. Changes in the resting membrane potential (RMP) of soleus muscles isolated from rats subjected to hypobaric hypoxia (HH) at a pressure equivalent to an altitude of 3000 m for 3 h. The measurements were carried out on muscles isolated 24 h after control or HH treatment followed by rat hindlimb suspension (HS) for 6 h. The measurements were completed in extrajunctional (**b**) and junctional (**c**) sarcolemmal regions. The number of muscles corresponds to the number of symbols. Two-way ANOVA followed by Bonferroni multiple comparisons test. ** *p* < 0.01— compared as indicated by horizontal bars.

Steady sarcolemmal depolarization is characteristic of motor dysfunction, such as in the hindlimb suspension (HS) model of skeletal muscle disuse. In the rat soleus muscle, HS-induced depolarization is predominantly the result of impaired α2 Na,K-ATPase function [34,35]. Our previous studies have shown that HS depolarized RMP of rat soleus muscle extrajunctional membrane to a similar extent (by ~3 mV) within 6–72 h of HS [34,35], which was accompanied by a significant shift of the initial part of the force–voltage relationship towards higher voltages, suggesting a decrease in membrane excitability [34]. This small but stable membrane depolarization is among the earliest disuse-induced remodeling events prior to atrophy or any change in contractility [34,35]. Thus, we tested whether HH could improve RMP in the soleus muscle following HS (Figure 9a), and 6 h of HS was chosen as the key time point in this study.

In these experiments, 24 h after HH, extrajunctional membrane hyperpolarized from −73.6 ± 0.4 mV in control soleus muscles to −76.2 ± 0.4 mV (*p* < 0.01) in hypoxia-treated muscles (Figure 9b). The same hyperpolarization was also observed 3 h after HH (data not shown), however, in these experiments, the rats were not subjected to subsequent HS. No HH-dependent changes in RMP were observed in the junctional membrane region (Figure 9c). Thus, HH similarly affected the sarcolemmal electrogenesis both in the respiratory diaphragm and in the postural soleus muscles.

In subsequent experiments, 24 h after exposure to control or HH treatment, rats were subjected to HS for 6 h. Similar to previous observations [35], 6 h of HS depolarized both extrajunctional and junctional membranes to approximately −70 mV. However, after HH, no HS-induced depolarization was observed in these membrane regions (Figure 9b,c), suggesting protection from this disruption of sarcolemmal electrogenesis.

## 3. Discussion

Maintaining a sufficient level of the RMP of sarcolemma is crucial for normal skeletal muscle function. Even a small but prolonged membrane depolarization leads to inactivation of sodium channels and suppression of membrane excitability that can disrupt the excitation–contracting coupling system [50,51]. Steady membrane depolarization due to loss of the Na,K-ATPase function is characteristic for skeletal muscle disuse of different nature [33,34,35,51]. A number of observations show that hypoxic preconditioning may protect diaphragm muscle function [13,15]. However, the ability of mild hypoxia to modulate the Na,K-ATPase to improve skeletal muscle electrogenesis has not been previously addressed. 

In this study, we tested this ability by subjecting rats to simulated high-altitude HH (3000 m above sea level for 3 h) using a pressure chamber. The novelty of our findings is that:

- A single HH did not affect serum corticosterone levels, cellular redox status, and LDH activity, which emphasizes the mild nature of the hypoxic state.
- In the diaphragm muscle, HH stably increased the α2 Na,K-ATPase isozyme electrogenic activity and hyperpolarized the sarcolemma within 24 h.
- These changes were accompanied by a steady decrease in the serum level of endogenous ouabain, a specific ligand of the Na,K-ATPase, as well as an increase in the TBARS production.
- HH increased the α2 Na,K-ATPase membrane abundance without changing its total protein content and did not affect the plasma membrane lipid-ordered phase.
- Intact diaphragm muscles exposed to the hypoxic incubation solution for 1 h showed a similar but reversible membrane hyperpolarization.
- In the soleus muscle, HH protected against disuse-induced membrane depolarization.

Our observations suggest that the prolonged modulating effects induced by HH require the triggering of long-term intracellular and/or circulating factors. Could this be the steady increase in TBARS production we are seeing? However, increased production of TBARS strongly correlates with inhibition of the Na,K-ATPase [52,53,54] and cannot explain our results. Notably, our observations do not contradict the increase in TBARS production. The adaptability of biological membranes to lipid peroxidation depends on their structure, and lipid raft domains enriched in sphingolipids and cholesterol are less sensitive to lipid peroxidation than other regions of the membrane [55]. Localization of the α2 Na,K-ATPase in lipid-ordered membrane phases and specific α2 Na,K-ATPase/cholesterol interactions have been previously proposed [47]. Thus, increased production of TBARS under mild oxidative stress was apparently unable to inhibit the α2 Na,K-ATPase localized in lipid rafts.

Lipid metabolism plays a major role in cell physiology, and its changes, which are part of hypoxic adaptation, remain poorly understood [56,57]. The activity of Na,K-ATPase is regulated by the lipid environment, which is realized through direct protein–lipid interactions and/or by influencing the physical properties of the lipid bilayer [45,46]. Localization of the α2 Na,K-ATPase in lipid-ordered membrane phases and specific α2 Na,K-ATPase/cholesterol interactions have been proposed [47]. It has been shown that 22-NBD-cholesterol is associated with lipid-disordered membrane regions and its distribution is opposite to cholesterol [48,49]. Thus, a decrease in 22-NBD-cholesterol fluorescence could reflect a corresponding redistribution of cholesterol between lipid-ordered and lipid-disordered membrane phases, which could increase the activity of α2 Na,K-ATPase [47]. However, no changes were observed in the lipid-ordered phase of the plasma membrane in this study.

Some data confirm an exceptionally high susceptibility of the α2 Na,K-ATPase subunit to oxidation [19]. Glucocorticoids are known to crosstalk with hypoxic signaling [43], and may be involved in protection against ischemia-reperfusion-induced injury [41]. Glucocorticoids were also shown to regulate the Na,K-ATPase expression in a variety of tissues [58] and 14 days of treatment with dexamethasone specifically increased the α2 subunit abundance in the rat diaphragm [59]. However, our study did not find significant changes in the level of corticosterone after 3 h HH.

The concentration of ouabain in the blood serum was stably reduced within 3–24 h after HH. Ouabain, a specific ligand of the Na,K-ATPase, has an endogenous circulating analog in the mammalian body. Increasing evidence indicates a broad therapeutic potential of circulating ouabain and the involvement of Na,K-ATPase/endogenous ouabain system in numerous physiological and pathological processes [24,29,30,31,32,60,61]. Notably, in rodents, the Na,K-ATPase α1 isozyme is relatively resistant to ouabain, while the α2 isozyme is more than 100 times more sensitive [32].

The concentration of endogenous ouabain in biological tissues was found to be extremely variable (for review [62]). This depends on the animal species, different physiological states, plasma or serum samples, the detection method, kit manufacturer, and many other circumstances. Indeed, the concentration of endogenous ouabain varies from 0.017 nM measured in murine plasma [63] to 3–4 nM, measured in fresh rat serum [64]. In this study, the concentration of endogenous ouabain in the serum of control rats was similar to those previously detected in the rat serum [64]. Elevated levels of endogenous ouabain have been shown in both physiological [65] and pathophysiological [24,29,30,31,32,60,61] conditions. Clinical [66] and experimental [42,67] hypoxic conditions as well as high altitude [68] are also considered as potential stimuli for the release of endogenous cardiotonic steroids. Conversely, in this study, our data provide the first evidence that short-term mild HH steadily **decreases** the circulating level of endogenous ouabain.

Notably, chronic exposure to hypoxia can affect the content of Na,K-ATPase in skeletal muscle. As was shown previously, exposure to hypoxia for 1–6 weeks at 380 Torr (~5500 m) increases the Na,K-ATPase content in the rat diaphragm muscle [12]. Conversely, downregulation of Na,K-ATPase has been shown in human vastus lateralis after 21 days at high altitude (6194 m) [69]. Changes in the electrogenic activity of α2 Na,K-ATPase may be associated with a change in its spatial distribution in the sarcolemma. In this study, HH did not affect the total α2 Na,K-ATPase protein content, but increased its membrane abundance. How can this be explained? A number of studies have reported that Na,K-ATPase undergoes internalization/endocytosis upon ouabain binding [70,71]. Chronic administration of ouabain (1 µg/kg/day for four days), which doubled circulating ouabain level [44,72], reduced the α2 Na,K-ATPase membrane content in the rat diaphragm and soleus muscles [73]. This was observed without changes in the total level of α2 Na,K-ATPase protein. This suggests that the elevated level of circulating ouabain modulates the α2 Na,K-ATPase subunit trafficking between its intracellular pool and the sarcolemma and/or its turnover in the membrane resulting in the reduced α2 Na,K-ATPase membrane abundance [73]. Thus, the HH-induced decrease in the level of circulating ouabain, as observed in this study, can cause an opposite effect—an increase in the α2 Na,K-ATPase membrane abundance, which we observed. This, at least in part, may be the reason for the HH-induced increase in the electrogenic activity of α2 Na,K-ATPase.

Ouabain, like other cardiotonic steroids, is a specific Na,K-ATPase inhibitor. However, a huge amount of data indicates the ability of ouabain to activate Na,K-ATPase at concentrations comparable to its endogenous level. It has been shown that ouabain at concentrations of 0.1–3 nM stimulates the α1 Na,K-ATPase in human endothelial cells [74,75] and in human kidney proximal tubule cells (0.001–0.1 nM) [72]. In the guinea pig myocytes, nanomolar ouabain (0.1–10 nM) stimulated α2 Na,K-ATPase, whereas higher concentrations resulted in pump inhibition [76]. In rat diaphragm muscle, ouabain at concentrations of 5–20 nM caused membrane hyperpolarization, which preceded depolarization at higher concentrations [44].

The mechanism of ouabain-induced Na,K-ATPase activation is still the subject of controversy. This stimulation was thought to result from the existence of two ouabain-binding sites with high (stimulatory) and low (inhibitory) affinities [76]. The existence of Na,K-ATPase in a form of (αβ)_2_ diprotomer with functionally different α subunits with different affinity for ouabain was also discussed [77]. The activating effect of ouabain can also be associated with a change in the [Na]_i_/[K]_i_ ratio [75].

Alternatively, it can be assumed that the implementation of the activating effect of ouabain requires a specific subcellular localization and the presence of certain molecular partners. For example, ouabain-mediated stimulation of the α1 Na,K-ATPase in renal cells requires a specific molecular environment such as Na,H-exchanger-1 [72] or angiotensin receptor type I, and can be modulated by an initial increase in intracellular Na^+^ concentration [78]. It is hypothesized that this Na^+^ accumulation triggered by ouabain binding enhances the translocation of Na,K-ATPase from the intracellular pool to the plasma membrane through an angiotensin/AT1R-dependent mechanism [78] and hence increases the membrane abundance of the enzyme. Although the presence of the Na,H-exchanger in mammalian skeletal muscles is well documented [79], the possibility of its involvement in the regulation of Na,K-ATPase in these cells has not been studied.

Another possibility is if accumulated Na^+^ could activate ouabain-free neighboring α2 Na,K-ATPase molecules. To operate in such manner, ouabain should induce Na^+^ accumulation in the subcellular spatially distinct compartments in places where the plasma membrane adjoins intracellular organelles (sarcoplasmic reticulum, etc.). In smooth and cardiac cells, these microdomains are characterized by specific clustering of the α2 Na,K-ATPase, Na,Ca-exchanger, Ca^2+^ channels, SERCA, ryanodine and IP_3_ receptors [80] and may include interactions with the microtubule network [81]. In the skeletal muscle, an analogue of such microdomains can be triadic junctions formed by T-tubules and terminal cisternae of the sarcoplasmic reticulum, where the above proteins are located, including the Na,Ca-exchanger [82] and the α2 Na,K-ATPase [27].

**Figure 10:**
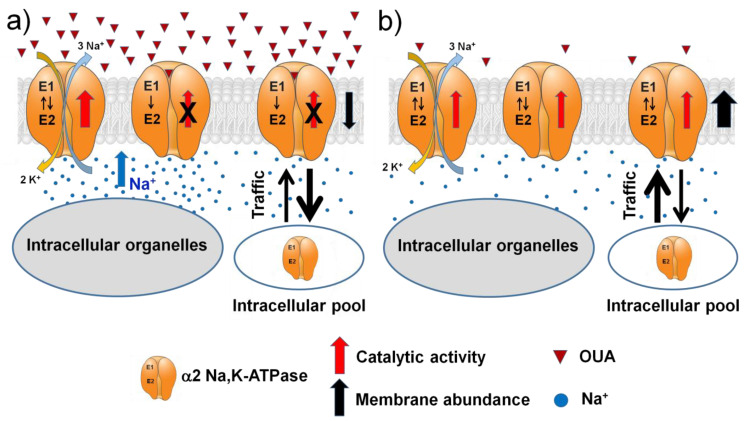
A schematic presentation of the dynamic regulation of α2 Na,K-ATPase transport activity by relatively high (**a**) and low (**b**) concentrations of circulating ouabain (see the text).

Considering all of the above, we hypothesized that circulating ouabain can regulate α2 Na,K-ATPase activity, modulating both catalytic activity and membrane abundance. This hypothetical mechanism also suggests the existence of distinct pools of α2 Na,K-ATPase regulated in different ways. With an increase in ouabain concentration, locally accumulated Na^+^ triggered by ouabain binding enhances the catalytic activity of ouabain-free neighboring α2 Na,K-ATPase molecules located in the subcellular spatially distinct membrane compartments with delayed diffusion. At the same time, ouabain modulates the α2 Na,K-ATPase trafficking between its intracellular pool and the sarcolemma resulting in the reduced membrane abundance. A decrease in the concentration of ouabain should produce opposite effects: a decrease in catalytic activity and an increase in the membrane abundance. These reciprocal changes may be the mechanism for dynamic regulation of overall α2 Na,K-ATPase transport activity by circulating ouabain and thus skeletal muscle electrogenesis. This hypothetical scheme is presented in the Figure 10.

It should be noted that further studies are needed to extrapolate the results of this study to subjects other than rats. However, unlike rodents, all Na,K-ATPase isozymes in other mammals, including humans, are relatively sensitive to ouabain [20,32,83,84] and cannot be differentiated pharmacologically using different concentrations of ouabain, as in this study. Therefore, other experimental approaches should be used, including the use of genetically engineered animals [32].

Finally, data on the effect of aging on RMP are scattered. Some data indicate that in human skeletal muscles there was no significant dependence of RMP on age, as well as differences between female and male subjects [85]. In skeletal muscles of rodents, the content of Na,K-ATPase decreases with age [86], but RMP of resting (non-contracting) muscles in aged and young rats did not differ [87]. However, in aged rats, increased depolarization with prolonged recovery and reduced adaptation of RMP to eccentric contractions were observed [87]. Thus, a greater dependence of aged muscles on physical activity can be assumed. This leaves open the question of whether short-term mild hypoxia affects older rats differently, and this can be regarded as a limitation of this work.

Altogether, it is known that hypoxia-associated effects are multidimensional in nature and dose-, time-, and use-dependent [19,56]. We suggest that our new findings are important for hypoxic therapy and might have an important implication in disuse-induced skeletal muscle pathology. Future studies will be required to identify the precise molecular mechanisms of the phenomenon we have revealed.

## 4. Materials and Methods

### 4.1. Animals

Experiments were performed on male Wistar rats aged 9–11 weeks (180–220 g). The impact of gender, age, and training status on Na,K-ATPase may interfere with each other and remain to be studied in detail [86,88,89]. In particular, primary and induced hypoxic tolerance and preconditioning are gender-dependent and endogenously modulated in females during the estrus cycle. Differences in hypoxic oxidative energy metabolism are part of differential tolerance, and this sex-related dependence should be considered [90]. In addition, a number of hormones such as the female sex hormone estradiol regulate the function of Na,K-ATPase [91]. Therefore, only male rats were used in this study.

Animals were housed in a temperature- and humidity-controlled room with food and water ad libitum. All procedures involving rats were performed in accordance with the recommendations for the Guide for the Care and Use of Laboratory Animals [92]. The experimental protocol met the requirements of the EU Directive 2010/63/EU for animal experiments and was approved by the Ethics Committee of St. Petersburg State University (issued 13 December 2017).

Simulated high-altitude exposure was performed in a decompression chamber (Tabai V-18, Osaka, Japan) maintained at a pressure equivalent to an altitude of 3000 m (530 Torr) above sea level at 22–24 °C. The chamber was continuously ventilated. Rats were subjected to HH for 3 h. Control group rats were maintained in the same chamber under normoxic conditions. Rats were euthanized 3 or 24 h after exposure to HH by intraperitoneal injection of a tribromoethanol overdose (750 mg/kg) followed by decapitation. The diaphragm muscle was isolated and mixed blood was collected. Freshly isolated diaphragm muscle was immediately used for electrophysiological measurements. Some muscles were also snap-frozen in liquid nitrogen and then stored at −80° C for later biochemical assays.

In separate experiments, 24 h after exposure to HH, rats were subjected to HS, widely used as an animal model of disuse that leads to progressive atrophy of postural skeletal muscles. The rats were subjected to HS individually in custom cages for 6 h, as described previously [93]. Control animals were not suspended. In these experiments, freshly isolated soleus muscles were immediately used for electrophysiological experiments.

### 4.2. Biochemical Analyses of Blood and Tissue Samples

Blood glucose or lactate levels were measured immediately before and after HH by applying drops of blood to appropriate chemically treated disposable “test-strips”. An electronic blood glucose meter (Accu-Chek Active, Roche Diabetes Care GmbH, Mannheim, Germany) and an electronic blood analyzer (Accutrend Plus, Roche Diagnostics GmbH, Mannheim, Germany) were used.

The serum level of ouabain was estimated using the ELISA Kit for ouabain (CEV857Ge, Cloud-Clone corp., Katy, TX, USA). Serum corticosterone level was estimated using the ELISA kit (AC-14F1, Xema, Saint Petersburg, Russia). The assay’s procedures were conducted in accordance with the manufacturer’s protocols and the light absorbance was measured at 450 nm with a spectrophotometric microplate reader SPECTROstar Nano (BMG Labtech, Ortenberg, Germany). The concentrations of ouabain and corticosterone were quantified based on the respective standard curves.

Redox status, lipid peroxidation, and LDH activity were estimated in the cytosolic fraction of muscle tissue.

The number of reduced thiol groups in cytosolic proteins were analyzed as a marker of redox status by adding 150 μL of 5,5′-dithiobis-(2-nitrobenzoic acid) derivatization solution (52.5 µg/mL) to 50 µL of the cytosolic fraction in 0.1 M potassium phosphate/1 mM ethylenediaminetetraacetic acid buffer (pH 7.4) [94]. The optical density was measured using a spectrophotometric microplate reader SPECTROstar Nano (BMG Labtech, Ortenberg, Germany) at 412 nm at room temperature. The concentration of thiol groups was quantified using a reduced glutathione standard curve and expressed as nmol of cysteine per mg of total protein.

Lipid peroxidation products in the muscle tissue lysate were estimated using the thiobarbituric acid reactive substances (TBARS) assay as described previously [95]. The optical density of TBARS was measured using a spectrophotometric microplate reader SPECTROstar Nano (BMG Labtech, Ortenberg, Germany) at 535 nm with a reference optical density wavelength of 600 nm at room temperature. The TBARS concentration was expressed in nmol per mg of total protein in the sample.

The enzymatic activity of LDH was assayed by measuring the decrease in optical density of the reduced NADH solution [96]. A reaction mixture containing (final concentrations) 0.3 mM Sodium Pyruvate and 16 μM NADH was added to 10 μL of samples. The optical density of NADH was measured using a spectrophotometric microplate reader SPECTROstar Nano (BMG Labtech, Ortenberg, Germany) at 340 nm at 37 °C for 10 min. The amount of NADH was determined using a NADH standard curve. LDH activity was calculated as nmol of NADH oxidized per minute per mg of total protein.

Total protein in the samples was measured using a Pierce Rapid Gold Bicinchoninic Acid Protein Assay Kit (Thermo Fisher Scientific, Waltham, MA, USA) according to the manufacturer’s protocol.

### 4.3. Membrane Potential Recording

The isolated diaphragm or soleus muscle with a nerve stump was placed in a chamber and continuously perfused with physiological solution containing (in mM): NaCl, 137; KCl, 5; CaCl_2_, 2; MgCl_2_, 2; NaHCO_3_, 24; NaH_2_PO_4_, 1; glucose, 11; pH 7.4. The solution was continuously gassed with 95% O_2_ and 5% CO_2_ and maintained at 28 °C. The RMP was recorded from the surface fibers using intracellular glass microelectrodes. The RMP recordings were made in the extrajunctional membrane regions within ~2 mm from visually identified terminal branches of the nerve, or directly near the nerve terminals, as described previously [28,97]. In each diaphragm muscle, RMPs were recorded from ~30–35 different fibers for each (junctional and extrajunctional) membrane region over a total time of about 5–10 min. In some experiments, diaphragm muscles isolated from intact rats were exposed to a normobaric hypoxia, followed by reoxygenation, as described previously [15]. Initial RMPs were recorded in the normal physiological solution bubbled with 95% O_2_ + 5% CO_2_. The muscles were then exposed to 60 min hypoxia (95 N_2_ + 5% CO_2_), followed by reoxygenation (95 O_2_ + 5% CO_2_).

Five diaphragm muscles were tested in experiments with hypobaric hypoxia, and 5–7 muscles were tested in experiments with normobaric hypoxia followed by reoxygenation. In the same way, in each of 7–8 soleus muscles, the RMP from ~25 different fibers was recorded. Thus, in all muscles, the total number of RMP measurements for each time point was at least 150. Then, for each muscle, the RMPs were averaged and this value was used for statistical analysis as a single experimental value and for the final mean calculation in the experimental series.

### 4.4. Measurement of the Na,K-ATPase Electrogenic Activity

The Na,K-ATPase electrogenic transport was determined by measuring ouabain-sensitive changes in the RMP. These changes are generated by electrogenic Na,K-ATPase transport. This is a previously characterized sensitive real-time assay to assess the Na,K-ATPase activity in intact skeletal muscle [28,51,98]. This method is based on more than 100-fold difference in affinities of the rodent α1 and α2 Na,K-ATPase isozymes for ouabain. Thus, in rat skeletal muscle, 1 μM ouabain inhibits the α2 isozyme without affecting the α1 isozyme, whereas 500 μM ouabain completely inhibits both isozymes [28,98]. For each muscle, the initial RMP value was recorded. Then, ouabain was sequentially added to the solution at concentrations of 1 and 500 μM. The electrogenic contribution of α2 isozyme was computed as the difference in mean RMP before and 30 min after the incubation with 1 µM ouabain. The electrogenic contribution of α1 isozyme was estimated as the difference in RMP with 1 µM ouabain and after 30 min incubation with 500 µM ouabain.

### 4.5. Western Blot Assays

Frozen muscles were homogenized in a lysis buffer containing (in mM): Tris, 20; NaCl, 150; Triton X-100, 1; EDTA, 5; tween-20, 0.1; protease inhibitors cOmplete mini tablets (Roche, Mannheim, Germany) (pH 7.6). After homogenization, samples were incubated on ice for 30 min, then sonicated and centrifuged for 15 min at 13,000 g at 4 °C (Eppendorf, Hamburg, Germany). Total protein content in the supernatants was measured with Pierce Rapid Gold Bicinchoninic Acid Protein Assay Kit (Thermo Fisher Scientific, Waltham, MA, USA) according to the manufacturer’s protocol using a spectrophotometric microplate reader SPECTROstar Nano (BMG Labtech, Ortenberg, Germany). Equal amounts of total protein were heated up for 10 min at 37° C with a 4× Laemmli buffer.

The total proteins were loaded on 10% Stain-Free gels and separated by SDS-PAGE (sodium dodecyl sulfate–polyacrylamide gel electrophoresis). Then, proteins were transferred to the PVDF membrane (Bio-Rad, Hercules, CA, USA). After blocking for 2 h with 5% skimmed milk at room temperature, the membranes were incubated overnight at 4 °C with 2.5% skimmed milk and monoclonal rabbit antibody against the α2 isoform (1:1000, MA5-37905, Thermo Fisher Scientific, Waltham, MA, USA). The next day, after intensive washing, the membranes were incubated for 45 min with 2.5% skimmed milk and horseradish-peroxidase-conjugated anti-rabbit secondary antibody (1:10,000, AB205718, Abcam, Eugene, OR, USA). The excess of antibody was removed by washing, and bound antibody was detected using an enhanced chemiluminiscence kit (Bio-Rad, Hercules, CA, USA). Band intensities were measured using a luminescence imager Chemi-Doc XRS + Imaging System (Bio-Rad, Hercules, CA, USA). Detected proteins were normalized using the Image Lab 6.1 Software (Bio-Rad, Hercules, CA, USA) to the total protein load in the same sample measured in the membrane prior to incubation with the antibody.

### 4.6. Confocal Microscopy Imaging

To identify the endplate membrane region, tetramethylrhodamine-α-bungarotoxin (α-BTX, Biotium, Fremont, CA, USA), a fluorescent-labeled specific ligand of the nAChRs was used. For selective imaging of the α2 Na,K-ATPase, a freshly isolated muscle was incubated for 15 min with physiological saline containing fluorescent-labeled specific ligands of the Na,K-ATPase (Bodipy-conjugated ouabain, 1 μM, a concentration that preferably affects the α2 Na,K-ATPase membrane pulls [28] and the nAChRs (rhodamine-conjugated α-BTX, 1 μM). The physiological solution was bubbled with 95% O_2_ and 5% CO_2_ and maintained at room temperature. Superficial regions of the muscle were imaged with a ×63, 1.3 NA objective using a Leica TCS SP5 confocal system configured for concurrent viewing of rhodamine and BODIPY fluorescence, as described previously [28].

Plasma membranes were also stained with fluorescent 22-NBD-cholesterol [22-(N-(7-nitrobenz-2-Oxa-1,3-diazol-4-yl)amino)-23,24-Bisnor-5-Cholen-3β-Ol, Molecular Probes], as reported previously [47]. 22-NBD-cholesterol was used as an environment-sensitive probe that localizes in the membrane’s interior. 22-NBD-cholesterol fluorescence intensity increases in response to phase changes in membrane lipids from rafts to nonraft fractions, and its distribution in phosphatidylcholine/cholesterol bilayers is opposite of that of cholesterol [48,49]. The spectral properties of 22-NBD-cholesterol are almost independent of phase state of the bilayer [49]. A stock solution of 22-NBD-cholesterol (10 mg/0.1 mL in ethanol) was prepared and used at a final concentration of 0.2 μM in a physiological solution, where muscles were incubated for 20 min.

Analysis of endplate fluorescence was performed in the region defined by α-BTX staining. The fluorescence intensity from the nAChRs and the α2 Na,K-ATPase was determined by using ImageJ software. Each region of the nAChRs labeling was outlined by hand using the “freehand” tool. The resulting outlines (masks) were then used to determine the localization of α2 Na,K-ATPase or 22-NBD-cholesterol fluorescence at the junctional (endplate) membrane region. The fluorescence intensity (arbitrary units) was defined as the ratio of total fluorescence intensity to the area of endplate. The mean endplate area and fluorescence intensities were assessed for each rat and then averaged. Extrajunctional fluorescence (arbitrary units) was calculated similarly for an area (~200 μm^2^) of sarcolemma outside of the α-BTX-positive region.

### 4.7. Materials

Chemicals were purchased from Sigma-Aldrich (Burlington, MA, USA).

### 4.8. Statistical Analysis

Data are presented as mean ± SEM. The statistical significance of the difference between means was evaluated using one- or two-way ANOVA followed by Bonferroni multiple comparisons test. Statistical analysis was performed using GraphPad Prism 8 software (GraphPad; San Diego, CA, USA). A probability value of *p* < 0.05 was considered statistically significant.

## Data Availability

The data that support the findings of this study are available from the corresponding author upon reasonable request.
